# Interferon regulatory factor 3 is a key regulation factor for inducing the expression of SAMHD1 in antiviral innate immunity

**DOI:** 10.1038/srep29665

**Published:** 2016-07-14

**Authors:** Shen Yang, Yuan Zhan, Yanjun Zhou, Yifeng Jiang, Xuchen Zheng, Lingxue Yu, Wu Tong, Fei Gao, Liwei Li, Qinfeng Huang, Zhiyong Ma, Guangzhi Tong

**Affiliations:** 1 Shanghai Veterinary Research Institute, Chinese Academy of Agricultural Sciences, Shanghai, 200241, P.R. China

## Abstract

SAMHD1 is a type I interferon (IFN) inducible host innate immunity restriction factor that inhibits an early step of the viral life cycle. The underlying mechanisms of SAMHD1 transcriptional regulation remains elusive. Here, we report that inducing SAMHD1 upregulation is part of an early intrinsic immune response via TLR3 and RIG-I/MDA5 agonists that ultimately induce the nuclear translocation of the interferon regulation factor 3 (IRF3) protein. Further studies show that IRF3 plays a major role in upregulating endogenous SAMHD1 expression in a mechanism that is independent of the classical IFN-induced JAK-STAT pathway. Both overexpression and activation of IRF3 enhanced the SAMHD1 promoter luciferase activity, and activated IRF3 was necessary for upregulating SAMHD1 expression in a type I IFN cascade. We also show that the SAMHD1 promoter is a direct target of IRF3 and an IRF3 binding site is sufficient to render this promoter responsive to stimulation. Collectively, these findings indicate that upregulation of endogenous SAMHD1 expression is attributed to the phosphorylation and nuclear translocation of IRF3 and we suggest that type I IFN induction and induced SAMHD1 expression are coordinated.

A number of recent studies have indicated the role of the sterile alpha motif and HD domain 1 (SAMHD1) protein in inhibiting virus infectivity. SAMHD1 blocks human immunodeficiency virus-1 (HIV-1) replication in myeloid-lineage cells^1^,^2^,^3^ and functions as a deoxynucleoside triphosphate (dNTP) triphosphohydrolase, which hydrolyzes dNTP pools to inhibit reverse transcription^4^. Besides HIV-1, SAMHD1 has been shown to play vital roles in STING-mediated apoptosis against human T-lymphotropic virus type 1 (HTLV-1) infection of primary human monocytes. SAMHD1 participates in the generation of reverse transcription intermediates (RTI) of HTLV-1. The RTIs complex with the innate immune sensor STING and initiate IRF3-Bax-directed apoptosis^5^. Moreover, SAMHD1 functions broadly to inhibit replication of DNA viruses. SAMHD1 could restrict replication of the HSV-1 DNA genome in differentiated macrophage cell lines, though the dNTP triphosphohydrolase activity^6^. Our previous study showed that proliferation of highly pathogenic porcine reproductive and respiratory syndrome virus (HP-PRRSV), an enveloped, single-stranded RNA virus, was efficiently blocked in MARC-145 cells over-expressing SAMHD1 and the antiviral effects of SAMHD1 on HP-PRRSV were through inhibition of HP-PRRSV replication^7^. Besides, the biological activity of SAMHD1 has been revealed. SAMHD1 may be a cellular regulator of long interspersed elements 1 (LINE-1) and LINE-1-mediated Alu/SVA retrotransposition^8^. Mutations in SAMHD1 are associated with the Aicardi–Goutières syndrome, an autoimmune disorder exemplified by irregular type I IFN responses. However, SAMHD1 mutations produced in the Aicardi–Goutières syndrome are defective in LINE-1 inhibition^9^. HIV-2 and certain strains of SIVsm that encode the Vpx protein utilized the CRL4^DCAF1^ and E3 ubiquitin ligase complex to recruit SAMHD1 for proteasome-dependent degradation^10^,^11^,^12^. SAMHD1 tetramerization is required for its biological activity and its expression is regulated by promoter methylation^13^,^14^. SAMHD1 expression induced by cytokines varies among different cell lines^3^. However, type I IFN treatment downregulates SAMHD1 phosphorylation, but does not upregulate endogenous SAMHD1 expression in human primary dendritic cells (DCs), CD4^+^ T lymphocytes, monocytes, and macrophages^15^,^16^. Human SAMHD1 is induced by IL-12/IL-18 in monocyte-derived macrophages (MDM), and by TNF-α in lung fibroblasts^17^,^18^. The specific regulatory mechanism by which SAMHD1 is upregulated remains unknown.

The innate immune response is an essential component of host defense against infections and plays an important role in shaping adaptive immunity^19^,^20^. Interferon blocks virus replication and inhibits virus dissemination and thus, many viruses have evolved strategies to evade IFN-induced antiviral responses^21^,^22^,^23^,^24^,^25^,^26^. The type I interferon signaling network initiates an antiviral response through host pattern recognition receptors (PRRs) which recognize pathogen-associated molecular patterns (PAMPs)^21^,^27^,^28^. Recognition of PAMPs by PRRs, such as Toll-like receptors (TLR3, TLR4, TLR7/8, TLR9) and the RIG-I-like receptor families (RIG-I and MDA5)^29^,^30^,^31^,^32^, with downstream signaling through IRF3, IRF7, and NF-κB leading to type I IFN production. The signaling of type I IFNs is activated by the interaction between IFN-α/β and their receptors on the cell surface, leading to the activation of Janus kinase (JAK) family. The JAK family phosphorylate the substrate proteins, signal transducers and activators of transcription (STAT) 1 and 2. Phosphorylated STAT1 and STAT2 work together with interferon regulatory factor 9 (IRF9) and translocate into the nucleus, resulting in the expression of IFN-stimulated genes (ISGs), which modulate the host immune responses^25^,^33^.

In the present study, in addition to confirming the previous findings that SAMHD1 expression can be upregulated in HeLa cells treated with type I IFN^15^, we provide further evidence that type I IFN treatment upregulates endogenous SAMHD1 expression in HEK293 cells, porcine macrophages and MARC-145 cells. We show that the TLR3 and RIG-I/MDA5 pathways participate in the regulation of SAMHD1 expression and find that IRF3 phosphorylation and nuclear translocation are critical aspects of SAMHD1 upregulation after IFN-α treatment and virus infection.

## Materials and Methods

### Cell culture and viruses

MARC-145 cells derived from an African green-monkey kidney cell line, HeLa cells and HEK293T cells were maintained in Dulbecco’s modified Eagle’s medium (DMEM, GIBCO). The human embryonic kidney cell line HEK293 was maintained in minimum essential medium (MEM, GIBCO). THP-1 cells were maintained in RPMI-1640 medium (GBICO). Primary porcine alveolar macrophages (PAMs) were prepared and maintained as previously described^34^. All cell lines were supplemented with 10% fetal bovine serum (FBS) at 37 °C with 5% CO_2_. THP-1 cells were differentiated with 50 ng/ml of phorbol 12-myristate 13-acetate (PMA) (Sigma-Aldrich). HP-PRRSV HuN4 strain was propagated at passage 5 in MARC-145 cells and inactivated by UV irradiation as described previously^34^,^35^,^36^. Briefly, the virus stocks were dispersed in 10-cm tissue culture dishes and placed directly under a UV lamp (20 W). Complete inactivation of the virus was confirmed by titration on MARC-145 cells. The Newcastle disease virus (NDV) strains Herts/33 and La Sota were obtained from the China Institute of Veterinary Drug Control (Beijing, China). Viruses were titrated and stored at −80 °C until used.

### Antibodies, reagents and plasmids construction

Rabbit monoclonal antibodies (mAb) against phospho-STAT1 (Tyr701), phospho-IRF3 (Ser396), IRF3 and polyclonal antibody against TRIF, as well as the RIG-I pathway antibody sampler kit were purchased from Cell Signaling Technology, and the IRF7 antibody was purchased from abcam. Polyclonal antibody against IRF3 were purchased from Active Motif and used for ChIP analysis. Anti-SAMHD1 antibody, anti-HA-Tag antibody produced in rabbit, anti-β-actin antibody, and an anti-FLAG M2 antibody produced in mouse were obtained from Sigma-Aldrich. All the primary antibodies could recognize the target proteins of the cells used in the study. The mouse monoclonal antibody against porcine SAMHD1 protein was prepared in our laboratory^37^. Mouse monoclonal antibodies recognizing NDV NP protein and porcine reproductive and respiratory syndrome virus (PRRSV) N protein were generous gifts from Dr. Chan Ding (Shanghai veterinary research institute, CAAS, Shanghai, China) and Shaoying Chen (Fujian academy of agricultural sciences, Fujian, China), respectively. Horseradish peroxidase (HRP)-conjugated anti-rabbit IgG and anti-mouse IgG were purchased from Jackson. Alexa Fluor 488-labeled goat anti-mouse antibody was purchased from Invitrogen. Universal type I interferon and porcine interferon alpha (mammalian) were obtained from PBL. Human, porcine IL-6 and TNF-α were purchased from R&D Systems. IRF3 phosphorylation inhibitor BX 795 was prepared with DMSO to 10 mM stock. IRF3 siRNA (h), IRF7 siRNA (h) and control siRNA-A were supplied by Santa Cruz Biotechnologies. Single-stranded RNA Double-Right (ssRNA DR) and its negative control ssRNA 41, poly (I:C) of RIG-I/MDA5 Ligand, poly (I:C) of TLR3 ligand, 5′ triphosphate double stranded RNA (5′ppp-dsRNA), and the Ready-made psiRNA-hSTAT1 kit were purchased from Invivogen. Dual-luciferase reporter assay system was purchased from Promega. NE-PER Nuclear and Cytoplasmic Extraction Reagents, Pierce Agarose ChIP Kit, and LightShift Chemiluminescent EMSA Kit were purchased from Thermo Fisher. IFN alpha-IFNAR-IN-1 were obtained from MedChem Express.

Human TRIF eukaryotic expression plasmid pCMV-HA-TRIF was constructed by inserting the TRIF CDS into pCMV-HA vector (Clontech), and human MAVS expression plasmid FLAG-MAVS was generated in our laboratory. The mammalian expression plasmids pFLAG-IRF3, pFLAG-IRF7 and pFLAG-TBK1 were constructed into mammalian expression vector p3 × FLAG CMV 7.1 (Sigma-Aldrich) by cloning the CDS sequences from the cDNA of HeLa cells using specific primers containing restriction enzyme cleavage sites (Supplementary Table 1). IRF3-5D, an active form of IRF3, and IRF7Δ247–467, a constitutively active form of IRF7 were constructed as previously described using pFLAG-IRF3, and pFLAG-IRF7 plasmids as templates^38^,^39^,^40^. STAT1 WT and STAT1 Y701F plasmids were purchased from Addgene. Amplification of the human SAMHD1 full-length promoter sequence was performed as previously described^14^ and was cloned into pGL3-Basic vector (Promega). Construction of mutated forms of the SAMHD1 promoter luciferase reporter plasmids (M1-M9) was done by PCR or overlap PCR and the reporter plasmid containing the predicted SAMHD1 full-length promoter region was used as a template. The primers are listed in Supplementary Table 1. The DNA sequences of the amplified fragments were confirmed using DNA sequencing and cloned into the pGL3-Basic vector with *Mlu* I and *Xho* I sites. All constructed plasmids were confirmed by DNA sequencing and enzyme digestion. pRL-TK luciferase reporter plasmid was purchased from Promega.

### Cell treatment, virus Infection, and western blot analysis

For interferon treatment, HeLa cells, HEK293 cell, THP-1 cells, MARC-145 cells, and PAMs in 60-mm dishes were grown to 70–80% confluence. Subsequently, all cells were treated with 1,000 U/mL universal type I Interferon and PAMs were treated with the same concentration of porcine interferon alpha or mock treated with the same medium. For other cytokine treatments, cells were treated with 100 ng/mL TNF-α or 50 ng/mL IL-6. The cells were then cultured for various times as indicated.

Growth-arrested MARC-145 cells and PAMs cultured in 60-mm dishes were infected with HuN4 or NDV, respectively, at an MOI of 1 or 5, or mock infected with the medium, and then incubated for indicated times.

To analyze whether inhibition of IRF3 phosphorylation and nuclear translocation would affect SAMHD1 expression, MARC-145 cells and HeLa cells were both pretreated with IRF3 phosphorylation inhibitor BX 795 for 2 h and then treated with IFN-α or NDV infection, and placed in serum-free medium containing fresh inhibitor and sustained for 16 h. PAMs were pretreated with BX 795 or IFN alpha-IFNAR-IN-1 for 2 h and then infected with HuN4 at an MOI of 5, or mock infected with the medium. DMEM containing DMSO was used for the mock treatment. After cells were infected or treated for the indicated time, the cells were then collected for western blot analysis as described previously^34^. The analysis of IRF3 dimer formation by Native SDS-PAGE was performed as previously described^41^.

### Quantitative Real-time RT-PCR and IFN-α expression determination

HeLa cells, HEK293 cells, THP-1 cells, MARC-145 cells, and PAMs were treated with IFN-α or infected with virus as indicated and then collected for RNA extraction. Total RNA isolation, cDNA synthesis, and real-time quantitative PCR analysis of SAMHD1 mRNA levels in treated cells were performed as previously described^7^,^15^,^26^,^42^. SAMHD1 gene transcript levels were analyzed using the 2^−ΔΔCT^ method^43^. Primers used for qPCR analysis are shown in Supplementary Table 1. The expression of IFN-α in PAMs was determined by ProcartaPlex Multiplex Immunoassays as described previously^44^.

### Transfection and luciferase reporter assay

HeLa cells, HEK293 cells, MARC-145 cells and PAMs were plated in 6-well culture plates at 70–80% confluence and transfected with poly (I:C), 5′-ppp dsRNA, ssRNA DR, ssRNA 41 at a concentration of 2 μg/mL or mock transfected by HiPerFect Transfection Reagent (Qiagen) for 24 h. The cell lysates were harvested and subjected to real-time RT-PCR and western blot analysis.

For shRNA transfection, MARC-145 cells and HEK293 cells were plated in 6-well culture plates at 70–80% confluence and transfected with 4 μg of shRNA targeting human STAT1 or shRNA control by FuGENE^®^ HD transfection reagent (Promega) for 48 h. Then the cells were selected using medium containing 50–150 μg/mL Zeocin (Life technologies) for 3 days until cell foci were identified. The selected cells were used for further study.

For luciferase reporter assay, the indicated plasmids were transfected into 5 × 10^4^ HeLa cells in 24-well culture plates along with pRL-TK as an internal reference control, using the FuGENE^®^ HD transfection reagent (Promega) according to the manufacture’s guidelines. After 24 h transfection, the cells were harvested and subjected to luciferase assay.

### Indirect immunofluorescence assay

HeLa cells grown on coverslips were transfected with IRF3, IRF3-5D, IRF7 and IRF7Δ247–467. Empty vector and mock transfections served as negative controls. At 48 h post-transfection, the cells were washed with PBS twice and then fixed with 4% paraformaldehyde for 15 min at room temperature. After washing three times in PBS, the cells were permeabilized by incubation with 0.5% Triton X-100 (Sigma-Aldrich) in PBS for 10 min, washed in PBS, and then blocked in 3% bovine serum albumin (BSA) for 30 min at 37 °C. Coverslips were then incubated with mouse anti-FLAG M2 monoclonal antibody (Sigma-Aldrich) in PBS at 37 °C for 1 h, washed three times in PBS, and then incubated with Alexa Fluor 488-labeled goat anti-mouse antibody (Invitrogen) at 37 °C for 30 min. The coverslips were stained with DAPI for 5 min at 37 °C, mounted in aqueous mounting medium (Sigma-Aldrich), and observed using confocal laser scanning microscopy.

### RNA interferon and complementation assay

HeLa cells were plated in 6-well culture plates and grown to 5 × 10^5^/well. Cells were transfected with 50 nM of IRF3 or IRF7 siRNA using X-tremeGENE siRNA Transfection Reagent (Roche) for 48 h and then incubated with type I IFN for 12 h. For IRF3 complementation, pFLAG-IRF3 was transfected into HeLa cells previously treated with IRF3 siRNA. 36 h post-transfection, cells were then treated with IFN-α for 12 h. Transfection efficiencies were quantified using western blot analysis.

### Chromatin Immunoprecipitation (ChIP)

HeLa cells were stimulated with IFN-α for 12 h and then processed for ChIP analysis using Pierce Agarose ChIP Kit, according to the manufacture’s instruction. Mock stimulated cells served as negative control. The ChIP analysis was performed as previously described^45^,^46^. Chromatin fragments were immunoprecipitated using normal rabbit IgG or IRF3 polyclonal antibody bound to beads. Real-time PCR analyses were performed using the primers (Supplementary Table 1) to amplify DNA sequences near −31–+19 region of SAMHD1 promoter.

### Electrophoretic Mobility Shift Assay (EMSA)

HeLa cells were transfected with poly (I:C) at concentration of 2 μg/mL or transfected with 2 μg of IRF3-5D for 24 h. Nuclear proteins were extracted from transfected HeLa cells using NE-PER Nuclear and Cytoplasmic Extraction Reagents. An oligonucleotide probe of −31–+19 or +69–+119 regions were prepared and 5′ end labeled with biotin. Detection of transcription factor-oligonucleotide complexes was performed using a LightShift Chemiluminescent EMSA Kit, according to the manufacture’s instruction.

### Statistical analysis

All results are representative of three independent experiments. Statistical analyses were performed with two-way ANOVA tests or Student’s *t*-test. Significant difference was defined as *p* < 0.05.

## Results

### Type I IFN upregulated SAMHD1 expression in porcine macrophages and MARC-145 cells

**Figure 1:**
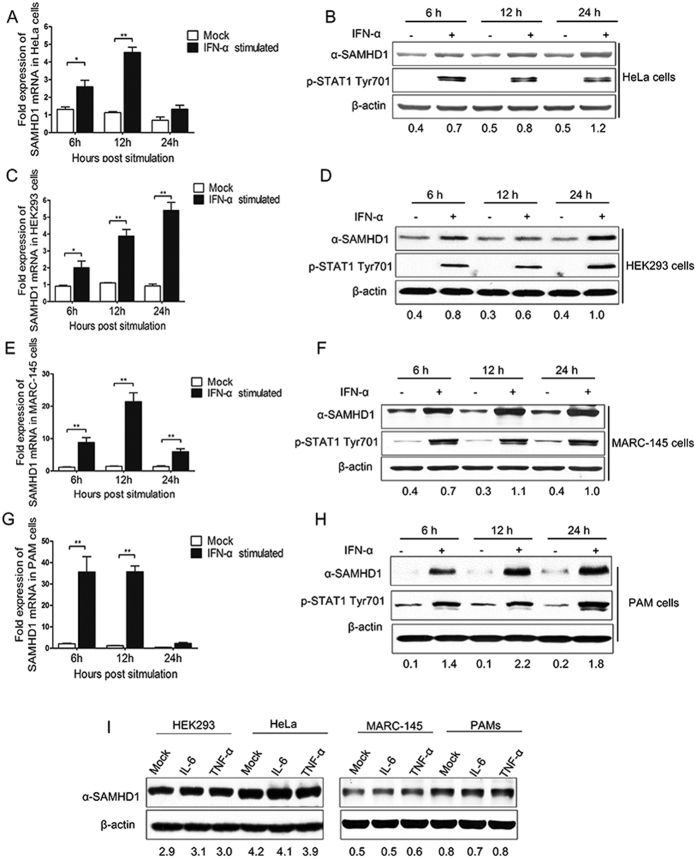
Type I interferon treatment upregulates SAMHD1 expression in human, monkey, and porcine cells. HeLa cells (**A**,**B**), HEK293 cells (**C**,**D**) and MARC-145 cells (**E**,**F**) were mock treated or treated with 1,000 U/mL of universal type I interferon. PAMs (**G**,**H**) were treated with porcine interferon alpha for 6–24 h. Samples were analyzed using RT-qPCR and Western blotting. (**I**) HeLa cells, HEK293 cells, MARC-145 cells and PAMs treated with IL-6 and TNF-α for 12 h. The expression of SAMHD1 in treated cells was analyzed. The fold change of SAMHD1 protein is expressed as densitometric units (Image J 1.45 s, National Institute of Health, USA) of the band normalized to the β-actin level, relative to the control. The error bar represents standard deviation from three independent experiments. The asterisks indicate a significant difference compared to mock treatment (*p < 0.05; **p < 0.01). Uncropped images of blots are shown in Supplementary Figure 1.

Previous studies showed that the levels of endogenous SAMHD1 protein in TCR-activated CD4^+^ T cells, monocytes, macrophages, dendritic cells (DCs), resting CD4^+^ T lymphocytes, and THP-1 cells were unaffected by type I IFN treatment^15^,^16^, while SAMHD1 protein levels significantly increased in HeLa cells and HEK293 cells treated with IFN-α (Fig. 1A–D) or with IFN-β^15^. To investigate changes in SAMHD1 expression as a function of type I IFN treatment in other cell lines, MARC-145 cells and PAMs (a primary porcine cell line) were treated with IFN-α. SAMHD1 mRNA and protein levels were both upregulated in the two cell lines compared to untreated cells (Fig. 1E–H). As expected, STAT1 phosphorylation was also enhanced by IFN-α treatment. These data suggest that SAMHD1 is a type I IFN inducible protein in MARC-145 cells and PAMs. Unlike in human myeloid-lineage cells, the amount of SAMHD1 in IFN-α treated PAMs increased (Fig. 1G,H), suggesting that SAMHD1 protein and mRNA are inducible and expressed in porcine macrophages after IFN-α treatment. Moreover, we also detected SAMHD1 expression in HeLa cells, HEK293 cells, MARC-145 cells and PAMs treated with proinflammatory cytokines, IL-6 and TNF-α for 12 h. Unlike IFN-α, the amount of SAMHD1 in IL-6 or TNF-α treated cells did not increase (Fig. 1I). Overall, these analyses confirm that type I IFN is also a key regulator for SAMHD1 expression in MARC-145 cells and porcine macrophages.

### SAMHD1 protein and mRNA expression were both enhanced by PRRSV infection in porcine macrophages, but not in MARC-145 cells

**Figure 2:**
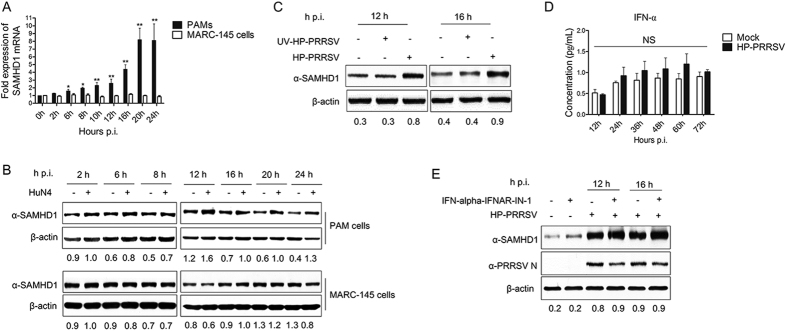
Changes in SAMHD1 expression in porcine macrophages and MARC-145 cells infected with HP-PRRSV. (**A**,**B**) PAMs and MARC-145 cells were infected with HP-PRRSV at an MOI of 5 and harvested at the indicated times. RT-qPCR (**A**) and western blot analysis of SAMHD1 expression (**B**) in HP-PRRSV infected cells. β-actin was used as a loading control. (**C**) The cell lysates of PAMs incubated with UV-inactivated HP-PRRSV for 12 h and 16 h were collected for western blot analysis of SAMHD1 protein expression. (**D**) The expression of IFN-α determined by ProcartaPlex Multiplex Immunoassays. PAMs were infected with HP-PRRSV for 12, 24, 36, 48, 60 and 72 h. The medium from mock infected cells served as a negative control. (**E**) PAMs were first pretreated with IFN-alpha-IFNAR-IN-1 or mock pretreated with medium containing DMSO for 2 h. The cells were then infected with the HP-PRRSV virus at an MOI of 5 or mock-infected with DMEM for 12 h and 16 h, respectively. The cell lysates were collected and analyzed by western blot. The fold change of SAMHD1 protein is expressed as densitometric units of the band normalized to the β-actin level relative to the uninfected control. The error bar represents standard deviation from three independent experiments. The asterisks indicate a significant difference compared to mock infection (NS, not significant: *p* > 0.05; *p < 0.05; **p < 0.01). Uncropped images of blots are shown in Supplementary Figure 2.

Our previous study showed that HP-PRRSV exhibited significant upregulation of SAMHD1 mRNA and protein expression in target cells (PAMs)^7^. In order to assess the role of PRRSV in the activation of SAMHD1 expression, we monitored the changes of SAMHD1 mRNA and protein in PAMs cells and MARC-145 cells. The cells were both infected with HP-PRRSV at an MOI of 5, harvested at the indicated times, and used for qRT-PCR and western blot analysis. Interestingly, SAMDH1 mRNA was gradually upregulated, whereas SAMHD1 protein was significantly increased at 12 h p.i. and continuously until 24 h p.i. in PAMs (Fig. 2A,B). In contrast, both the expression of SAMHD1 mRNA and protein showed no significant variation in MARC-145 cells infected with HP-PRRSV (Fig. 2A,B). SAMHD1 expression has no change in PAMs incubated with the UV-inactivated PRRSV when compared to cells infected with native viruses (Fig. 2C). As an immunosuppressive virus, PRRSV inhibits the expression of type I IFNs in host cells^26^. Previous results showed that IFN-α was also a positive regulator of SAMHD1 expression in PAMs and MARC-145 cells, which are permissive cells of PRRSV (Fig. 1E–H). In order to eliminate the effect of IFN-α on SAMHD1 expression in the context of viral infection, we further analyzed the expression of IFN-α in PRRSV infected PAMs. The cell culture supernatants of PAMs infected with HP-PRRSV were collected at the indicated times and then used for Multiplex Immunoassays according to the manufacturer’s instructions. Interestingly, the expression of IFN-α in HP-PRRSV infected PAMs was significantly inhibited (Fig. 2D), which was consistent with previous studies^47^. Furthermore, we treated PAMs with IFN alpha-IFNAR-IN-1, which is an inhibitor of the interaction between IFN-α and IFNAR and exerts immunosuppressive activity by the direct interaction with IFN-α and specifically inhibits IFN-α responses. We then analyzed the expression of SAMHD1 in HP-PRRSV infected PAMs. As expected, upregulation of SAMHD1 expression induced by HP-PRRSV was not inhibited in PAMs treated with IFN alpha-IFNAR-IN-1 (Fig. 2E). Taken together, these results indicate that SAMHD1 expression is upregulated by HP-PRRSV in the infection of PAMs, which is different from what was observed in MARC-145 cells, and type I IFN production is not required for the induction of SAMHD1 expression in porcine macrophages infected with HP-PRRSV.

### TLR3 and RIG-I signaling pathways contribute to SAMHD1 expression

**Figure 3:**
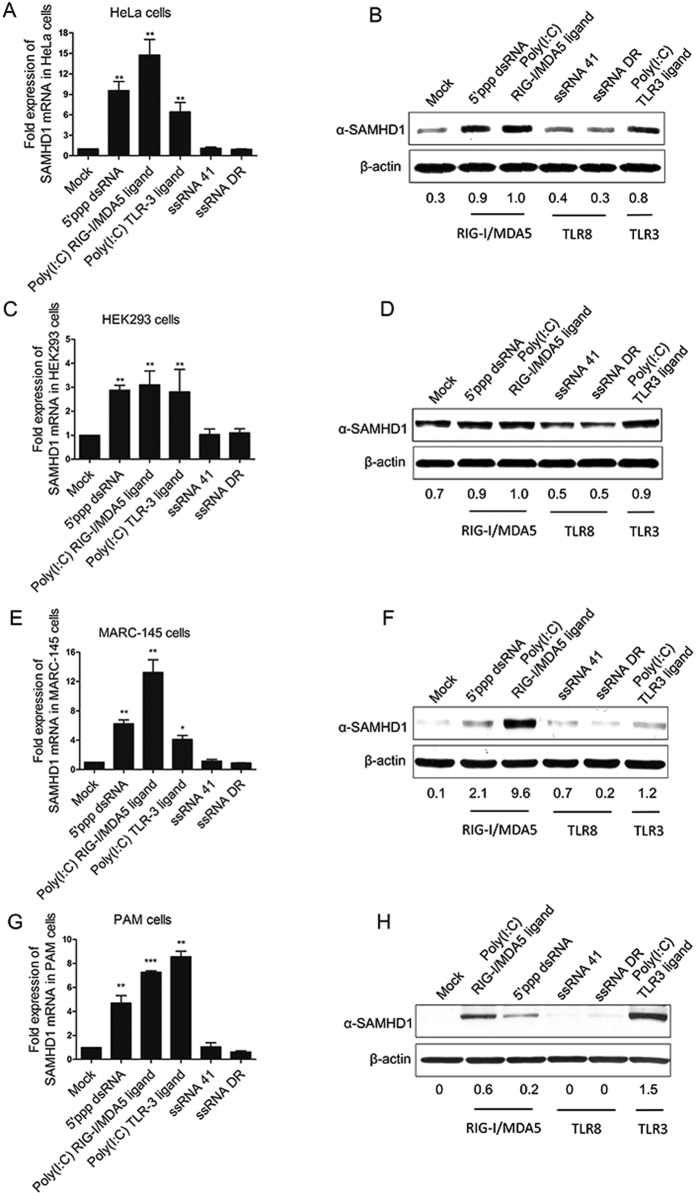
TLR3 and RIG-I/MDA5 agonists upregulate SAMHD1 expression in HeLa cells, HEK293 cells, MARC-145 cells and porcine macrophages. HeLa cells (**A**), HEK293 cells (**B**), MARC-145 cells (**C**) and PAMs (**D**) were treated with the indicated chemicals at a final concentration of 2 ug/mL and then analyzed using RT-qPCR and Western blotting. The error bar represents standard deviation from three independent experiments. The asterisks indicate a significant difference (*p* < 0.01) compared to mock transfection. Equal whole cell lysates were subjected to western blotting for analysis of SAMHD1 expression. β-actin was used as a loading control. The fold change of SAMHD1 is expressed as densitometric units of the band normalized to the β-actin level, relative to the control. Uncropped images of blots are shown in Supplementary Figure 3.

SAMHD1 is expressed in both cycling and resting cells, but its induction by various stimuli can differ. Our previous study showed that HP-PRRSV upregulated SAMHD1 expression in PAMs, and was independent of IFN-α (Fig. 2) , but did not induce expression of SAMHD1 in infected MARC-145 cells (Fig. 2A,B). We speculate that the antiviral innate immunity participates in the upregulation of SAMHD1. Next, we used different agonists to determine which stimuli were able to induce SAMHD1 expression in primary and immortal cell lines. Poly (I:C) induces the activation of the TLR3 and RIG-I/MDA5 signaling pathway and 5′ppp-dsRNA is a synthetic ligand for RIG-I. ssRNA DR is a potent immunostimulant that is recognized by TLR7/8^48^,^49^. SAMHD1 expression was significantly upregulated both by poly (I:C) and 5′ppp-dsRNA transfection at 24 h in HeLa cells (Fig. 3A,B), MARC-145 cells (Fig. 3E,F) and in PAMs (Fig. 3G,H). But SAMHD1 expression was only slightly enhanced by poly (I:C) and 5′ppp-dsRNA transfection in HEK293 cells (Fig. 3C,D). Neither transfection of ssRNA DR nor ssRNA 41, a negative control for the ssRNA DR, induced SAMHD1 expression at the time-points investigated. These results indicate that SAMHD1 upregulation is part of an early innate immune responses triggered by TLR3 and RIG-I/MDA5 stimulation.

### Overexpression of TRIF and MAVS induces SAMHD1 expression

**Figure 4:**
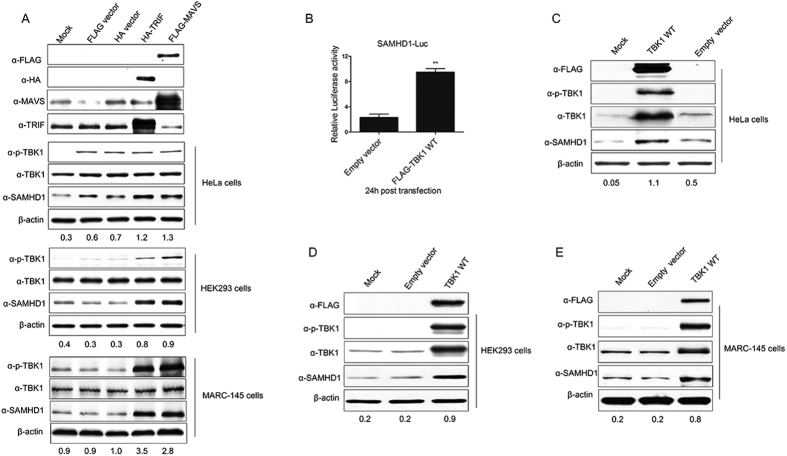
MAVS, TRIF and the downstream adaptor TBK1 upregulates SAMHD1 expression. (**A**) HeLa cells, HEK293 cells and MARC-145 cells were transfected with MAVS or TRIF expression plasmids and analyzed using Western blotting. (**B**) TBK1 activation upregulates SAMHD1 expression and promoter luciferase activity. SAMHD1 promoter luciferase activity was measured in HeLa cells. Cells were transfected with TBK1 or empty vector for 24 h and luciferase reporter activity was measured. Results are expressed as the fold-increase of luciferase activity in TBK1 overexpression cells. The error bars represent standard deviation from three independent experiments and asterisks indicate a significant difference (**p < 0.01), compared to empty vector transfection. Western blotting analysis of SAMHD1 expression in HeLa cells (**C**), HEK293 cells (**D**) and MARC-145 cells (**E**) transfected with FLAG-tagged TBK1 WT and empty vector, respectively. The results are representative of three independent experiments. Expression levels of SAMHD1 compared to β-actin are shown. Uncropped images of blots are shown in Supplementary Figure 4.

In response to stimulation with dsRNA, TLR3 recruits the downstream adaptor protein TRIF and RIG-I/MDA5 interacts with the mitochondrial adaptor protein MAVS (also known as IPS-1, CARDIF, or VISA), both of which play pivotal roles in antiviral innate immunity^50^,^51^,^52^. In order to investigate the roles of these TLR3 and RIG-I/MDA5 adaptors in the upregulation of SAMHD1 expression, we then transfected both HA-TRIF and FLAG-MAVS into HeLa, HEK293 and MARC-145 cells. Overexpression of the target proteins in each respective cell type was confirmed by anti-HA, anti-FLAG, and TRIF or MAVS specific antibodies, and transfection of TRIF and MAVS significantly upregulated SAMHD1 expression in the three cell lines, compared with the empty vector transfection (Fig. 4A). Thus, TRIF and MAVS that mediate activation of cellular intrinsic immune responses, play important roles in the upregulation of SAMHD1 expression. The results further confirmed that TLR3 and RIG-I/MDA5 induce SAMHD1 expression through TRIF and MAVS, respectively.

### TBK1 activation is required for SAMHD1 expression

TRIF and MAVS both activate the downstream kinases TANK-binding kinase 1 (TBK1) and IκB kinase (IKK-ε), which in turn activates the transcription factors IRF3 and NF-κB to initiate type I IFN production^53^,^54^,^55^,^56^. Our previous results indicated that SAMHD1 expression was upregulated by overexpression of TRIF and MAVS in HeLa, HEK293 and MARC-145 cells. TBK1, downstream of MAVS and TRIF, was also activated (Fig. 4A). TBK1 activated and slightly upregulated the expression of SAMHD1 only in HeLa cells transfected with empty vector (Fig. 4A). A previous study showed that DNA transfection of mammalian cells triggered cGAMP production, which bounds to STING, leading to the activation of IRF3^47^. We speculate that HeLa cells may be more sensitive to DNA transfection than other cell lines. Thus, we hypothesized that the downstream kinase TBK1 might take part in SAMHD1 activation after stimulation. TBK1 is phosphorylated on Ser172 within its activation loop, which is necessary for its ability to phosphorylate IRF3^58^. We initially compared the SAMHD1 promoter luciferase activity in HeLa cells transfected with wild-type TBK1. As compared with the empty vector transfection, SAMHD1 promoter luciferase activity was significantly increased in cells transfected with wild-type TBK1 (Fig. 4B). TBK1 overexpression elevated SAMHD1 protein levels in HeLa, HEK293 and MARC-145 cells, as compared with the empty vector control (Fig. 4C–E). Taken together, as an essential kinase engaged downstream of MAVS and TRIF, TBK1 is vital in upregulating SAMHD1 expression after activated by upstream adaptors.

### IRF3 plays a direct role in SAMHD1 transcriptional regulation

**Figure 5:**
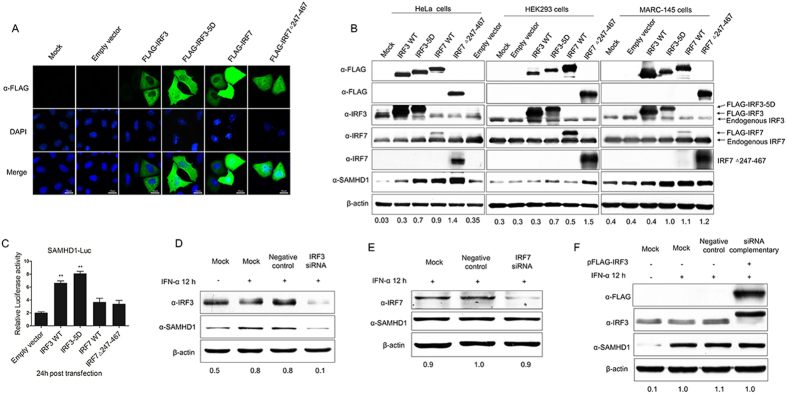
Effect of IRF proteins on SAMHD1 expression and promoter luciferase activity. (**A**) Immunofluorescence analysis of nuclear localization of IRF proteins. HeLa cells were plated onto cover slips and transfected with 2 μg of FLAG-tagged IRF3, IRF7 and its mutants, or mock transfected with empty vector DNA for 48 h. Cells were stained with mouse monoclonal antibody to FLAG (green) and nuclei were stained using DAPI (blue). Image quantification is for three independent experiments. Scale bars represent 10 μm. (**B**) HeLa cells, HEK293 cells and MARC-145 cells were transfected with FLAG-tagged IRF3, IRF3-5D, IRF7, IRF7Δ247–467, or empty vector. Expression levels of SAMHD1 compared to β-actin are shown. (**C**) Analysis of SAMHD1 promoter luciferase activity in HeLa cells transfected with IRF3, IRF7, and mutants for 24 h. Results are expressed as fold increase of luciferase activity in IRF3 and IRF3-5D overexpression cells. The error bars represent data from three independent experiments. The asterisks indicate a significant difference (**p < 0.01; *p < 0.05). (**D**,**E**) SAMHD1 upregulation is impaired in the absence of IRF3. HeLa cells were transfected with 50 nM IRF3 or IRF7 siRNA for 48 h, and then treated with 1, 000 U/mL IFN-α for 12 h. Cells lysates were subjected to western blotting to analyze IRF3 and SAMHD1 expression. (**F**) HeLa cells treated with IRF3 siRNA were complemented by transfecting 2 μg of pFLAG-IRF3 for 36 h and then treated with IFN-α for 12 h. IRF3 and SAMHD1 expression were confirmed using specific antibodies. Uncropped images of blots are shown in Supplementary Figure 5.

Innate immune responses are initiated by activating TLRs and RLRs signaling pathways, leading to the nuclear translocation of a set of transcription factors, including NF-κB, AP-1, and IRFs. Once activated, these transcription factors translocate to the nucleus, and cooperatively regulate the transcription of their target genes to induce the transcription of IFNs^50^. SAMHD1 is a strictly non-shuttling nuclear protein and the SAMHD1 expression induced by the innate immune signaling cascades has not been discussed^59^. We further assessed the effect of two important interferon regulatory factors (IRF3 and IRF7) on SAMHD1 expression, which are downstream effectors of TBK1 and key activators of type I interferon genes. IRF3 WT, IRF7 WT, and constitutively-active mutants of these proteins (IRF3-5D and IRF7Δ247–467) were transfected into HeLa, HEK293 and MARC-145 cells to investigate the inducible expression of endogenous SAMHD1. Overexpressed IRF3 was found mainly in the cytoplasm, whereas IRF3-5D, IRF7 and IRF7Δ247–467 were translocated into nucleus in absence of stimulation (Fig. 5A). As expected, the expression of endogenous SAMHD1 was upregulated in HeLa, HEK293 and MARC-145 cells transfected with IRF3-5D, IRF7 WT and IRF7Δ247–467. However, the overexpression of IRF3 WT had little influence on stimulating SAMHD1 expression (Fig. 5B). An IRF3-5D mutant, in which serine or threonine residues at positions 396, 398, 402, 404, and 405 were replaced by phosphomimetic aspartic acid residues, activated the IFN response^38^,^60^. IRF7 is another member of the IRF family, which is associated with the IFN response. Unlike IRF3, IRF7 WT over-expression stimulated the interferon gene expression and its constitutively active form, IRF7Δ247–467, activated the IFN-α response^39^. We speculate that the constitutively-active forms of IRFs may upregulate SAMHD1 expression. We then investigated which IRFs play a direct role in SAMHD1 transcriptional regulation. SAMHD1 promoter luciferase activity was assessed in HeLa cells transfected with the IRF constructs. SAMHD1 promoter activity was activated by IRF3 and IRF3-5D, as compared with IRF7 and IRF7Δ247–467 (Fig. 5C).

To confirm further the role of IRF3 and IRF7 in upregulation of SAMHD1 expression, HeLa cells were transfected with 50 nM IRF3 or IRF7 siRNA to reduce IRF3 or IRF7 expression. At 48 h post-transfection, cells were stimulated with 1,000 U/mL IFN-α for 12 h. IRF3 protein abundance was significantly reduced in HeLa cells transfected with IRF3 siRNA, with concomitant reduction of SAMHD1 levels after IFN-α treatment (Fig. 5D). But, SAMHD1 protein expression was not significantly affected by reducing the IRF7 protein abundance (Fig. 5E). Moreover, complementation with an IRF3 expression plasmid restored SAMHD1 abundance (Fig. 5F). Overall, the results indicate that only activated forms of IRF3 and IRF7 can induce SAMHD1 expression. Moreover, the activated form of IRF3 may directly induce SAMHD1 expression.

### SAMHD1 expression is independent of the JAK-STAT pathway

**Figure 6:**
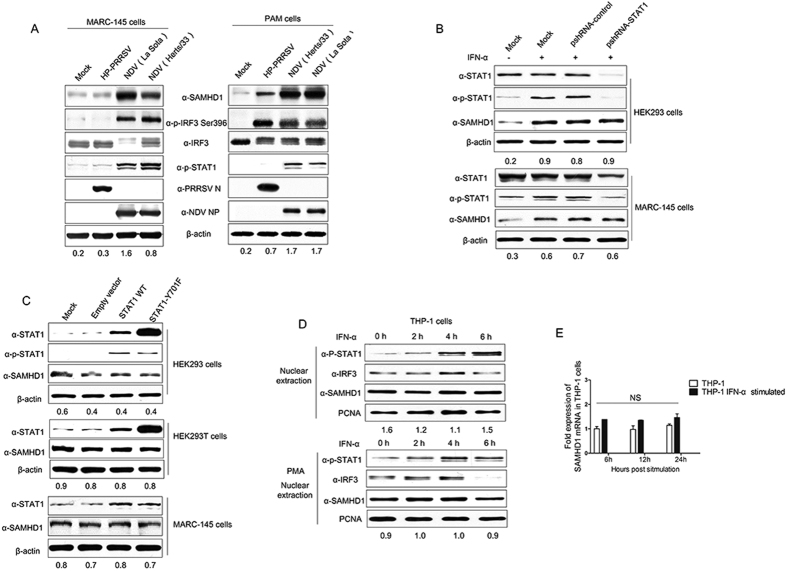
Virus infection or type I IFN mediated upregulation of SAMHD1 is independent of STAT1, but dependent upon IRF3. (**A**) MARC-145 cells and PAMs were infected or mock infected with PRRSV or NDV at an MOI of 1 for 16 h. The levels of SAMHD1 expression, phosphorylation of IRF3, and STAT1, were analyzed using Western blotting. (**B**) HEK293 cells and MARC-145 cells were transfected with psiRNA vector expressing shRNA targeting STAT1 gene for 36 h, then the cells were cultured in selective medium 50–150 μg/mL Zeocin (Life technologies) for 3 days until cell foci were identified. The cells were treated with IFN-α for 12 h. STAT1 and SAMHD1 expression were analyzed using Western blotting. (**C**) HEK293 and MARC-145 cells transfected with STAT1 WT or STAT1 Y701F plasmids were analyzed for SAMHD1 expression at 48 h post-transfection using western blotting. (**D**) THP-1 cells were either non-differentiated or differentiated overnight with 50 ng/ml of PMA, and then treated with 1,000 U/ml human IFN-α for 0–6 h. Nuclear proteins were extracted and the nuclear translocation of STAT1, IRF3, and SAMHD1 expression were detected using Western blotting. PCNA was used as a protein loading control. Expression levels of SAMHD1 compared to β-actin or PCNA are shown. (**E**) THP-1 cells were mock treated or treated with 1,000 U/mL IFN-α for the indicated times. Quantitative RT-PCR was performed using SAMHD1 specific primers and all data was normalized to β-actin (NS, not significant: *p* > 0.05). Uncropped images of blots are shown in Supplementary Figure 6.

Type I IFN binding to type I IFNs receptors activates the JAK-STAT pathway. STAT1 has been shown to be an important component of JAK-STAT signaling pathway. In order to assess the role of STAT1 in the IFN-mediated activation of SAMHD1 expression, MARC-145 and PAM cells were respectively infected with HP-PRRSV or NDV at an MOI of 1 for 16 h and then harvested for western blot analysis. In MARC-145 cells, NDV infection significantly up-regulated SAMHD1 expression, but HP-PRRSV did not (Fig. 6A). Similarly, NDV infection induced STAT1 phosphorylation, but HP-PRRSV did not (Fig. 6A). In PAMs, both HP-PRRSV and NDV infection obviously up-regulated SAMHD1 expression, together with the enhancement of IRF3 phosphorylations, but only NDV infection induced the STAT1 phosphorylation in PAMs (Fig. 6A). To confirm the role of STAT1 in SAMHD1 expression, we further analyzed the expression of SAMHD1 induced by IFN-α in MARC-145 and HEK293 cells, in which the STAT1 expression was silenced by shRNA targeting STAT1 gene. The expression of STAT1 and the phosphorylation of STAT1 were abrogated by shRNA, but SAMHD1 expression was still upregulated by IFN-α treatment in MARC-145 and HEK293 cells (Fig. 6B). Meanwhile, overexpression of STAT1 WT or its mutant STAT1 Y701F did not result in increased levels of SAMHD1 in HEK293, HEK293T and MARC-145 cells (Fig. 6C), suggesting that stimulation of SAMHD1 expression does not require STAT1 expression.

In Fig. 6C, although the phosphorylation of STAT1 was obviously upregulated in HEK293 cells overexpressing STAT1 WT or its mutant STAT1 Y701F, it did not result in increased levels of SAMHD1. A previous study showed that SAMHD1 expression was not upregulated by IFN-α in THP-1 cells and other human primary cells, but that the phosphorylation of STAT1 was significantly increased^16^. We further analyzed the phosphorylation and nuclear translocation of STAT1 in THP-1 cells and differentiated THP-1 cells treated with PMA. Both in cycling cells (THP-1 cells) and macrophages (PMA treated THP-1 cells), IFN-α treatment promoted the phosphorylation and nuclear translocation of STAT1, but did not upregulate SAMHD1 expression (Fig. 6D). The levels of SAMHD1 mRNA were examined over a time course from 6 to 24 h post-treatment with IFN-α. SAMHD1 mRNA level was at a steady state throughout the time course (Fig. 6E). These data support earlier results suggesting that type I IFN does not upregulate SAMHD1 expression in human macrophages and immortal cell lines^16^, and suggest that upregulation of endogenous SAMHD1 expression is independent of the phosphorylation and nuclear translocation of STAT1 and JAK-STAT signal pathway.

### IRF3 phosphorylation and nuclear translocation activity are required to upregulate SAMHD1 expression

**Figure 7:**
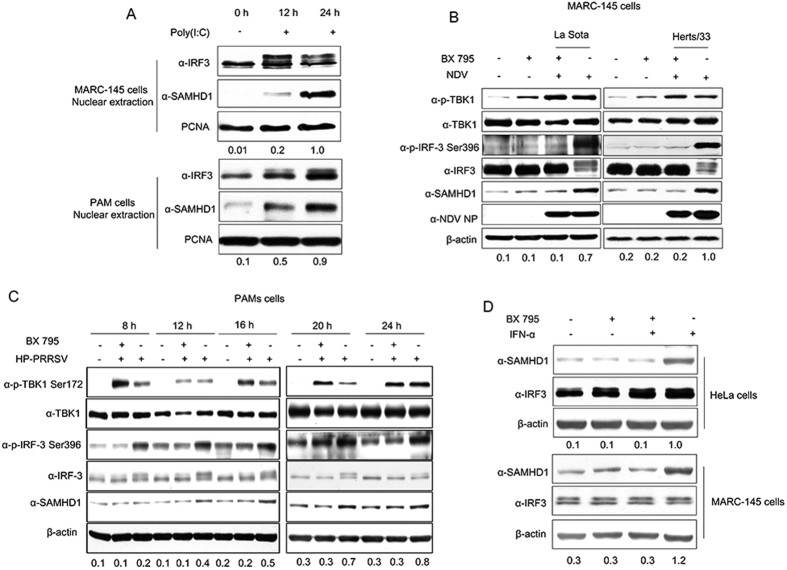
Phosphorylation and nuclear translocation of IRF3 plays important roles in inducible expression of SAMHD1. (**A**) MARC-145 cells and PAMs were transfected with poly (I:C) for 12 h and 24 h. Nuclear proteins were extracted and the nuclear translocation of IRF3 and SAMHD1 expression were analyzed using Western blotting. PCNA was used as a loading control. (**B**) MARC-145 cells were pretreated with 2 μM BX 795 for 2 h, and then infected with NDV at an MOI of 1 for 16 h together with or without the inhibitor. (**C**) The upregulation of SAMHD1 expression was inhibited in PAMs infected with PRRSV by BX 795. PAMs were pretreated with 1 μM BX 795 for 2 h and then infected with HP-PRRSV at an MOI of 5 for 8, 12, 16, 20 and 24 h. BX 795 was present throughout the duration of infection. HP-PRRSV infected PAMs were used as a positive control, and the untreated cells served as a negative control. Changes in TBK1, IRF3 phosphorylation, and SAMHD1 expression were evaluated using the specific monoclonal antibodies as indicated. (**D**) HeLa and MARC-145 cells were pretreated with 2 μm BX 795 for 2 h, and then treated with 1,000 U/mL IFN-α, in the presence of the inhibitor for 12 h or left untreated. Expression levels of SAMHD1 compared to β-actin or PCNA are shown. Uncropped images of blots are shown in Supplementary Figure 7.

**Figure 8:**
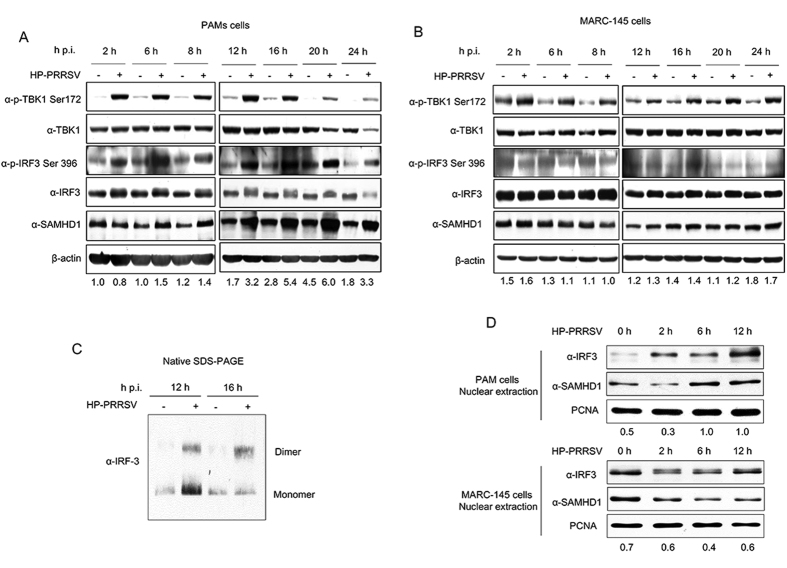
Activation of IRF3 together with SAMHD1 upregulation in virus infected cells. PAMs (**A**) and MARC-145 cells (**B**) infected with HP-PRRSV at an MOI of 5 were harvested at indicated times, and used for western blot analysis. Changes in TBK1, IRF3 and SAMHD1 expression were detected by western blotting. β-actin was used as a loading control. (**C**) Cell lysates of PAMs infected with HP-PRRSV were collected at 12 h and 16 h p.i. and analyzed by Native SDS-PAGE to visualize dimers of IRF3. (**D**) MARC-145 cells and PAMs were infected with HP-PRRSV for 2, 6 and 12 h. Nuclear proteins were extracted and the nuclear translocation of IRF3 and SAMHD1 expression were analyzed using Western blotting. PCNA was used as a loading control. Expression levels of SAMHD1 compared to β-actin or PCNA are shown. Uncropped images of blots are shown in Supplementary Figure 8.

Having shown the roles of JAK-STAT signal pathway in inducing SAMHD1 expression, we next investigated the relationship between the nuclear translocation of IRF3 and SAMHD1 protein expression. In Fig. 6D, the nuclear translocation of IRF3 was also largely unaffected in THP-1 cells. Then, we detected the relationship between IRF3 nuclear translocation and SAMHD1 expression in MARC-145 cells and PAMs, stimulated by poly (I:C). As expected, the IRF3 nuclear translocation was enhanced by poly (I:C) treatment, together with an increase in SAMHD1 abundance (Fig. 7A). Next, we blocked the nuclear translocation, phosphorylation, and transcriptional activity of IRF3 using BX 795^61^, in MARC-145 cells infected with NDV, which is a good activator of IRF3 phosphorylation and nuclear translocation^41^. The results showed that IRF3 phosphorylation was inhibited by BX 795 treatment and SAMHD1 failed to increase in abundance in MARC-145 cells after NDV infection (Fig. 7B). We further assessed SAMHD1 expression in PAMs treated with BX 795 and then infected by HP-PRRSV. As expected, the upregulation of SAMHD1 and phosphorylated IRF3 protein expression by PRRSV infection was significantly inhibited in the presence of BX 795 treatment (Fig. 7C). Moreover, we added additional IFN-α to explore the expression of SAMHD1 in MARC-145 cells and HeLa cells treated by BX 795. The results showed that IFN-α failed to induce SAMHD1 expression in the presence of BX 795 treatment (Fig. 7D). To further confirm a role for IRF3 in inducing the expression of SAMHD1, we analyzed the phosphorylation of TBK1 and IRF3, downstream targets of RIG-I/MDA5 and TLR3, in PRRSV infected PAMs and MARC-145 cells. The results showed that the TBK1 was significantly phosphorylated both in PAMs and MARC-145 cells during PRRSV infection (Fig. 8A,B). Meanwhile, IRF3 phosphorylation was upregulated and the dimer of IRF3 was obviously increased in PAMs infected with PRRSV, together with the upregulation of SAMHD1 (Fig. 8C). On the contrary, phosphorylation of IRF3 was not significantly induced in MARC-145 cells (Fig. 8B). Moreover, we also detected IRF3 nuclear translocation and SAMHD1 expression in MARC-145 cells and PAMs infected with HP-PRRSV. The two cell types were both infected with HP-PRRSV for 2 h, 6 h and 12 h at an MOI of 5, and then harvested for nuclear protein extraction. SAMHD1 and IRF3 protein in nuclear protein extractions of MARC-145 cells infected with HP-PRRSV showed no changes in protein levels across the time course. In contrast, increases in SAMHD1 and IRF3 protein levels were observed in nuclear protein extractions of PAMs infected with HP-PRRSV (Fig. 8D). The data further confirmed that IRF3 may predominantly regulate SAMHD1 expression independent of type I IFNs in antiviral innate immunity.

### Activated IRF3 induces SAMHD1 expression through binding to the SAMHD1 promoter

**Figure 9:**
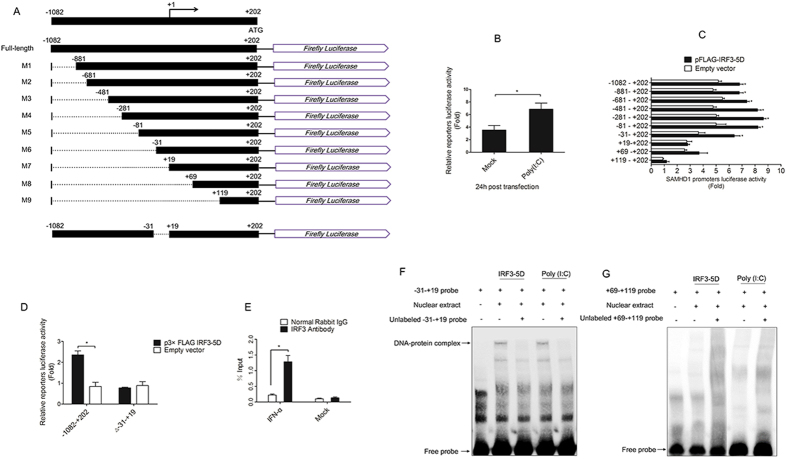
Identification of cis-acting elements responsible for SAMHD1 promoter activation by activated IRF3. (**A**) Schematic representation of pGL3-Basic SAMHD1 promoter full-length (FL) and 5′ deletions (M1 to M9), and deficient mutations in the full-length promoter (Δ-31–+19). (**B**) HeLa cells were transfected with pGL3-Basic SAMHD1 promoter full-length construct together with a *Renilla* luciferase reporter vector (pRL-TK-luc) for 6 h and then stimulated with poly (I:C). The cells were harvested at 24 h post-transfection and analyzed for dual-luciferase activity. (**C**,**D**) IRF3-5D and a series of promoter reporter constructs together with pRL-TK-luc were co-transfected into HeLa cells. Samples were collected 24 h post-transfection and analyzed for dual-luciferase activity. (**E**) HeLa cells were treated with or without IFN-α for 12 h and then processed for ChIP analysis. Antibody to IRF3 or Normal rabbit IgG was used to precipitate chromatin-bound IRF3. DNA sequences amplified near −31–+19 are shown. The asterisks indicate a significant difference (*p < 0.05, **p < 0.01) compared to empty vector transfection. Error bars represent the standard deviation (SD) calculated from three independent experiments. (**F**,**G**) EMSA was performed with −31–+19 probe, +69–+119 probe and with nuclear extracts from HeLa cells transfected with IRF3-5D or poly (I:C) after 24 hours. 20-fold molar excess of unlabeled probes served as competitor probes.

Activated IRF3 enters the nucleus and binds to the IFN-stimulated responsive element (ISRE, as known as the PRD I and III) to induce type I IFN responses^62^. IRF3 activated SAMHD1 promoter activity and induced the expression of endogenous SAMHD1 (Fig. 5). We further explored the transcriptional regulation of the human SAMHD1 gene by IRF3 using a luciferase assay. The full-length SAMHD1 promoter sequence was selected for the promoter studies and the luciferase activity of full-length SAMHD1 promoter was enhanced by poly (I:C) (Fig. 9B), which was consistent with a previous study that showed poly (I:C) could induce the expression of SAMHD1^63^. A series of SAMHD1 promoter deletion mutants (named M1-M9) were cloned into the pGL3-Basic luciferase vector (Fig. 9A). Sequential 5′ deletions from nucleotides −1,082 to −31 (M1 to M6) did not substantially alter constitutive or inducible luciferase expression after IRF3-5D induction, compared to the full-length promoter (Fig. 9C). By contrast, the M7-M9 deletion constructs displayed a lower or undetectable basal luciferase activity and were not inducible by IRF3-5D, suggesting that the minimal promoter region responsive to IRF3 induction lies between positions −31 to +19 (Fig. 9C). Luciferase activity was reduced after deleting the −31 to +19 region, as compared with the full-length promoter (Fig. 9D). Collectively, these findings suggest that activated IRF3 induces upregulation of SAMHD1 expression by binding to the SAMHD1 promoter. To confirm these findings, a ChIP assay was performed using an IRF3 specific antibody and primers encompassing the −31 to +19 region of the SAMHD1 promoter. HeLa cells were stimulated with IFN-α for 12 h and then processed for IRF3 ChIP. The rabbit IgG and mock treated HeLa cells served as negative controls. Equivalent DNA was performed to real-time quantitative PCR, and the results showed that the −31 to +19 (from start codon) primer amplified specific DNA bands treated by IRF3 antibody which were more intense than normal IgG, and we found no specific bands enhanced by IRF3 antibody treatment in mock treated HeLa cells (Fig. 9E). The ChIP results were confirmed by normal PCR (data not shown), and DNA sequencing of PCR products revealed that the sequence was matched to the SAMHD1 promoter sequence. Moreover, we designed a set of DNA probes used in EMSA to identify whether the −31 to +19 region is a binding site of IRF3. The regions of −31 to +19 and +69 to +119 were labeled with biotin at the 5′ end. The nucleoproteins of HeLa cells transfected with IRF3-5D or poly (I:C) for 24 h were extracted. In Fig. 9F, DNA-protein complexes were observed in the nuclear extracts incubated with the −31–+19 probe. Conversely, no DNA-protein complexes were observed in the nuclear extracts incubated with the +69–+119 probe (Fig. 9G). Unlabeled −31–+19 and +69–+119 oligonucleotides served as additional controls and were added to the binding reactions. The DNA-protein complex was competed out by the −31–+19 competitor (Fig. 9F). These data further confirm that induction of SAMHD1 by activated IRF3 is likely achieved through IRF3 binding to the –31 to +19 base region of the SAMHD1 promoter.

## Discussion

Cross-talk between innate immune signaling pathways and restriction factors can skew host responses towards either tolerance or defense against invading pathogens^64^. As an immunosuppressive RNA virus, HP-PRRSV infection inhibits the production of type I IFNs, both *in vivo* and *in vitro*^65^. Previous studies showed that PRRSV infection significantly blocked IRF3 phosphorylation and nuclear translocation induced by dsRNA or Sendai virus (SeV)^25^,^66^. While further studies showed that Nsp1 did not block phosphorylation and nuclear translocation of IRF3, but inhibited IRF3 association with CREB-binding protein (CBP) in the nucleus and modulated the induction of type I interferon in MARC-145 and HeLa cells^67^,^68^. Moreover, nuclear translocation of STAT1/STAT2 was also blocked in PRRSV-infected cells, leading to inhibition of the expression of ISGs in PAMs, and indicates that PRRSV infection inhibits the IFN signaling^25^. Both studies did not explore the specific signaling pathways related to type I IFNs production. However, SAMHD1 expression is significantly upregulated in PAMs infected with HP-PRRSV (Fig. 2A,B). Conversely, the expression of SAMHD1 is not upregulated in MARC-145 cells infected with HP-PRRSV (Fig. 2A,B). We speculated that SAMHD1 upregulation is part of an early cellular response to infection that is independent of interferon. We found that TLR3 and RIG-I/MDA5 agonists, but not TLR7 and TLR8 agonists, induced SAMHD1 upregulation in PAMs (Fig. 3). The phosphorylation and dimerization of IRF3 were increased in PAMs infected with HP-PRRSV (Fig. 8). We speculate that the phosphorylation and nuclear translocation of IRF3 were not inhibited, but the production of type I IFN was blocked by inhibiting IRF3 association with CBP in the nucleus in PAMs infected with HP-PRRSV. Due to antibodies limitation, we first detected the TBK1 and IRF3 changes in PRRSV infected cells. TLR3 expression has no changes both in MARC-145 cells and PAMs, and the expression of MDA5 was significantly increased in PAMs (data not shown). However, antibodies detecting RIG-I and MDA5 in MARC-145 cells or RIG-I in PAMs did not work. Surprisingly, the RIG-I/MDA5/TBK1/IRF3 signaling cascade was significantly activated in PAMs infected by HP-PRRSV, and the dimerization of IRF3 was increased together with the expression of SAMHD1 (Fig. 8), but the expression of IFN-α was inhibited (Fig. 2D). PRRSV blocks the RIG-I/MDA5 and TLR3 signaling cascades by inhibiting the phosphorylation of IRF3 in MARC-145 cells, resulting in no change in the expression of SAMHD1 (Fig. 8).

SAMHD1 expression may be induced by multiple stimuli^3^. Previous studies have shown that SAMHD1 is not sufficient to block virus infection in proliferating cells, due to loss of its activity after phosphorylation at Thr592 by cyclin A2/CDK1^16^,^69^. Surprisingly, SAMHD1 expression was not sensitive to IFN-α in activated CD4^+^ T cells, MDDCs, resting CD4^+^ T cells, monocytes, and macrophages, but the phosphorylation at Thr592 could be regulated by type I interferon. Type I IFN only upregulated SAMHD1 protein levels in HEK 293T and HeLa cell lines. However, SAMHD1 mRNA levels were increased at 6 h and 12 h post-treatment with IFN-α in DCs. In addition, a TLR9 agonist upregulated SAMHD1 mRNA level in peripheral blood mononuclear cells^15^,^16^,^70^.

mRNA quantification often does not reflect the increased protein expression as measured by western blotting. Our data show that SAMHD1 expression and transcription of mRNA are both upregulated by HP-PRRSV infection^7^ and by IFN-α in porcine macrophages (Fig. 1G,H). Stimulation of macrophages with a combination of IL-12 and IL-18 prevented or blocked productive infection by HIV-1 and the expression levels of SAMHD1, at both mRNA and protein levels, were increased in IL-12/IL-18 monocyte-derived macrophages (MDMs). SAMHD1 overexpression was not dependent on IFN-γ, implying that additional regulation mechanisms may modulate SAMHD1 function^18^. We compared SAMHD1 expression levels following treatment with IFN-α or virus infection in multiple cell lines and found that type I IFN could also regulate the expression of SAMHD1 in MARC-145 cells and porcine macrophages. Our data show that TLR3 and RIG-I/MDA5 agonists upregulate SAMHD1 expression. As an IKK family kinase, TBK1 plays central roles in inducing the production of IFNs. To explore how the PRRs activated SAMHD1 expression after stimulation, wild type TBK1 was transfected into HEK293 and HeLa cells. TBK1 WT transfection significantly upregulated SAMHD1 expression. SAMHD1 expression was upregulated only in cells transfected with IRF3-5D (IRF3 active form), IRF7 and IRF7Δ247-467, which have different abilities to induce IFN-α production^60^. Transfection of HEK293 and HEK293T cells with the IRF3-5D, IRF7 and IRF7Δ247–467 caused a release of type I IFNs as measured by ELISA, but IRF3 WT had no effect on IFN production (data not shown). Virus infection results in the activation of various transcription factors by specific phosphorylation. The transcription factors, IRF3 and IRF7 are activated through dimerization in cytoplasm and directly translocated into nucleus and are responsible for the production of type I IFNs^71^. Besides, IRFs are shown to be essential regulators of other target genes of primary response. Tetherin expression (also known as BST-2), another IFN-inducible host innate immunity restriction factor, is upregulated by IRF7^15^,^60^. Although IRF7 and IRF7Δ247–467 transfection induced endogenous SAMHD1 expression, the SAMHD1 promoter luciferase activity was unaffected. Because transfection of IRF3 and IRF3–5D activated the SAMHD1 promoter luciferase activity, but an inactive form of IRF3 did not upregulate SAMHD1 expression, we suspect that phosphorylation of IRF3 is a key factor for SAMHD1 expression.

RIG-I signaling through MAVS also activates the inhibitor of NF-κB (IκB) kinase (IKK) kinase complex, resulting in phosphorylation and subsequent proteasomal degradation of IκBα, therefore releasing active NF-κB dimers and allowing their nuclear translocation and transactivation of NF-κB-dependent genes^72^. PRRSV and NDV infection are good models for exploring innate immune signaling. PRRSV infection inhibits the expression of type I IFNs in host cells by interfering with the RIG-I pathway, IRF3 phosphorylation, and JAK/STAT pathway activation^24^,^25^,^26^,^73^, but still activates the NF-κB pathway^74^,^75^,^76^. NDV is an efficient inducer of type I interferon, through NF-κB activation, phosphorylation and dimerization of IRF3 and its V protein targets STAT1 for proteasome-mediated degradation^77^,^78^,^79^,^80^. In the present study, we use the two viruses as models to investigate the mechanisms underlying SAMHD1 transcriptional regulation in the antiviral immunity. In *vivo*, PAMs are primary target cells for PRRSV. In *vitro*, MARC-145 cells provide an important tool for the study of PRRSV replication. HeLa and HEK293 cells have high transfection efficiency and are widely used in exploring the cell signaling transduction pathways. At first, we use these cells lines to explore the regulation pathways of SAMHD1 through transfection. Then, we sought to demonstrate whether SAMHD1 induction is one of the early cellular responses to viral infection. Cells infected with HP-PRRSV or NDV were collected to further evaluate the virus-induced cellular antiviral responses associated with SAMHD1 transcript regulation. In HP-PRRSV infected MARC-145 cells, SAMHD1 expression was inhibited and phosphorylation of IRF3 and STAT1 were both inactivated. NDV infection eupregulated SAMHD1 expression and phosphorylation of IRF3 and STAT1. It will be important to determine whether inhibiting IRF3 phosphorylation might impair the expression of SAMHD1 induced by NDV infection or IFN-α treatment. BX 795 inhibits the phosphorylation, nuclear translocation, and transcriptional activity of IRF3, but the canonical NF-κB signaling pathway is unaffected^61^. As expected, BX 795 inhibited the induction of SAMHD1 expression by NDV or IFN-α treatment. Furthermore, in human monocyte-derived macrophages, neither addition of poly (I:C) to the cell culture medium nor transfection of poly(I:C) induced formation of detectable IRF3 dimers or nuclear translocation^81^. These data may explain why SAMHD1 expression is not regulated by IFN-α in human primary dendritic cells (DCs), CD4^+^ T lymphocytes, monocytes, and macrophages^15^,^16^. Taken together, the upregulation of SAMHD1 expression is mainly regulated through IRF3, but not through STAT1 or NF-κB signaling.

SAMHD1 has been shown to be an intrinsic host factor to block replication of various viruses in myeloid-lineage cells, and SAMHD1 expression induced by cytokines or virus infections varies among different cell lines^3^. Previous studies explored the phenomenon that SAMHD1 could be induced by different stimulus. However, the specific regulatory mechanism by which SAMHD1 is upregulated remains unknown. Efficient and robust induction of type I IFN is an important innate antiviral immune response. But, prolonged IFN will develop in the opposite direction when they become extreme. So, there is an important negative feedback mechanism in the regulation of type I IFN production in virus-infected cells. IRF3 has been established as an essential factor required for the production of type I IFN after virus infection. Our findings reveal that SAMHD1 could be activated by the same signals that trigger type I IFN production, via TLR3 and RIG-I/MDA5 signaling pathways, soon after viral infection. Upregulation of SAMHD1 in response to virus-induced IRF3 activation would ensure that host cells maintain SAMHD1-mediated virus restriction mechanisms together with the type I IFN responses. The important roles of IRF3 in the regulation of SAMHD1 expression adds to our understanding of the innate immune antiviral response and the delicate regulatory mechanism that control it.

In summary, the demonstration that phosphorylation of IRF3 contributes to inducing upregulation of SAMHD1 expression has important consequences for understanding host innate immunity and in the future management of virus infection. Although some degree of success has been achieved in managing host immune responses to reduce the burden of viral infection^4^,^6^,^82^, the battle between host and virus has yet to be explored. The interaction between human SAMHD1 and host proteins or virus has been discovered and is still ongoing^10^,^11^,^12^. Our data suggest that upregulation of SAMHD1 is an important aspect of the anti-viral response and future characterization of this pathway of host innate immune responses against virus may suggest efficacious strategies for vaccine and antiviral development.

## Additional Information

**How to cite this article**: Yang, S. *et al.* Interferon regulatory factor 3 is a key regulation factor for inducing the expression of SAMHD1 in antiviral innate immunity. *Sci. Rep.* **6**, 29665; doi: 10.1038/srep29665 (2016).

## Supplementary Material

Supplementary Figure 1

Supplementary Figure 2

Supplementary Figure 3

Supplementary Figure 4

Supplementary Figure 5

Supplementary Figure 6

Supplementary Figure 7

Supplementary Figure 8

Supplementary Figure 9

Supplementary Figure 10
