# Supplementary material for: Interferon regulatory factor 3 is a key regulation factor for inducing the expression of SAMHD1 in antiviral innate immunity
Source: Sci Rep. 2016 Jul 14;6:29665. doi: 10.1038/srep29665 (PMC4944147; doi:10.1038/srep29665)
Supplement: Supplementary Figure 1 [file srep29665-s1.pdf]

Interferon regulatory factor 3 is a key regulation factor for inducing the expression of SAMHD1 in antiviral innate immunity

Shen Yang, Yuan Zhan, Yanjun Zhou, Yifeng Jiang, Xuchen Zheng, Lingxue Yu, Wu Tong, Fei Gao, Liwei Li, Qinfeng Huang, Zhiyong Ma , Guangzhi Tong[*]

Shanghai Veterinary Research Institute, Chinese Academy of Agricultural Sciences, Shanghai, 200241, PR China

Supplementary Table and Figure Legends

Supplementary Table 1: Primers used in this study.

Supplementary Figure 1: Uncropped blot images in Figure 1.

Supplementary Figure 2: Uncropped blot images in Figure 2.

Supplementary Figure 3: Uncropped blot images in Figure 3.

Supplementary Figure 4: Uncropped blot images in Figure 4.

Supplementary Figure 5: Uncropped blot images in Figure 5.

Supplementary Figure 6: Uncropped blot images in Figure 6.

Supplementary Figure 7: Uncropped blot images in Figure 7.

Supplementary Figure 8: Uncropped blot images in Figure 8.
