# Supplementary material for: Interferon regulatory factor 3 is a key regulation factor for inducing the expression of SAMHD1 in antiviral innate immunity
Source: Sci Rep. 2016 Jul 14;6:29665. doi: 10.1038/srep29665 (PMC4944147; doi:10.1038/srep29665)
Supplement: Supplementary Figure 2 [file srep29665-s2.pdf]

Interferon regulatory factor 3 is a key regulation factor for inducing the expression of SAMHD1 in antiviral innate immunity

Shen Yang, Yuan Zhan, Yanjun Zhou, Yifeng Jiang, Xuchen Zheng, Lingxue Yu, Wu Tong, Fei Gao, Liwei Li, Qinfeng Huang, Zhiyong Ma , Guangzhi Tong*

Shanghai Veterinary Research Institute, Chinese Academy of Agricultural Sciences, Shanghai, 200241, PR China

Supplementary Table 1. Primers used in this study			
**Purpose**	**Name**	**Sequence (5'to 3')**	**Product Size**
**IRF3 amplification**	FLAG-IRF3 forward	CC*AAGCTT*GGAACCCCAAAGCCACGGAT	1,284 bp
	FLAG-IRF3 reverse	GC*TCTAGA*TCAGCTCTCCCCAGGGCCCTG	
**IRF7 amplification**	FLAG-IRF7 forward	CC*AAGCTT*ATGGCCTTGGCTCCTGAGA	1,512 bp
	FLAG-IRF7 reverse	GC*TCTAGA*CTAGGCGGGCTGCTCCAGC	
**TBK1 amplification**	FLAG-TBK1 forward	TT*GCGGCCGC*GATGCAGAGCACTTCTAATCAT	2,190 bp
	FLAG-TBK1 reverse	CG*GGATCC*CTAAAGACAGTCAACGTTGCG	
**Real-time PCR**	Pig SAMHD1 forward	TGGCAAATAAAAGAAATGGC	125 bp
	Pig SAMHD1 reverse	CTCACAGACACGGGCGAAT	
	Pig β-actin forward	CCGCACCACTGGCATTGTC	208 bp
	Pig β-actin reverse	CTCCTTGATGTCCCGCACG	
	MARC SAMHD1 forward	CACACTCGCAACTCTTTACACC	134 bp
	MARC SAMHD1 reverse	CGATACTTTTTTCCTCCAGCAC	
	MARC β-actin forward	GAGCGGGAAATCGTGCGTGACAT	187 bp
	MARC β-actin reverse	GGAAGGAAGGTTGGAAGAGAGCC	
**ChIP assay**	ChIP primer forward	GTTCTGCTTCTAGCCACG	145 bp
	ChIP primer reverse	TGACAGTTGAGCCCTTCG	
**Luciferase assay**	M1 forward primer	CG*ACGCGT*ACTTAATTCATTTAGTATT	
	M2 forward primer	CG*ACGCGT*GCTGGGAAGGTGTGTTGGC	
	M3 forward primer	CG*ACGCGT*CCACTTTCCTTCCTCTGGG	
	M4 forward primer	CG*ACGCGT*GTTTTCCTCTTCACTGGGA	
	M5 forward primer	CG*ACGCGT*CCTCAGTTCTGCTTCTAGC	
	M6 forward primer	CG*ACGCGT*TGGCGGGATTGATTTGAGG	
	M7 forward primer	CG*ACGCGT*AGCCCAAGGCAAGAGCCGC	
	M8 forward primer	CG*ACGCGT*AGTGAGCCTGCGCAGGAGG	
	M9 forward primer	C*GACGCGT*GCCAGGGCCCTGCTGTCAG	
	M reverse primer	CC*CTCGAG*GGCTACACCTGGCGTCCGG	
