# Supplementary material for: Interferon regulatory factor 3 is a key regulation factor for inducing the expression of SAMHD1 in antiviral innate immunity
Source: Sci Rep. 2016 Jul 14;6:29665. doi: 10.1038/srep29665 (PMC4944147; doi:10.1038/srep29665)
Supplement: Supplementary Figure 3 [file srep29665-s3.pdf]

Interferon regulatory factor 3 is a key regulation factor for inducing the expression of SAMHD1 in antiviral innate immunity

Shen Yang, Yuan Zhan, Yanjun Zhou, Yifeng Jiang, Xuchen Zheng, Lingxue Yu, Wu Tong, Fei Gao, Liwei Li, Qinfeng Huang, Zhiyong Ma , Guangzhi Tong*

Shanghai Veterinary Research Institute, Chinese Academy of Agricultural Sciences, Shanghai, 200241, PR China

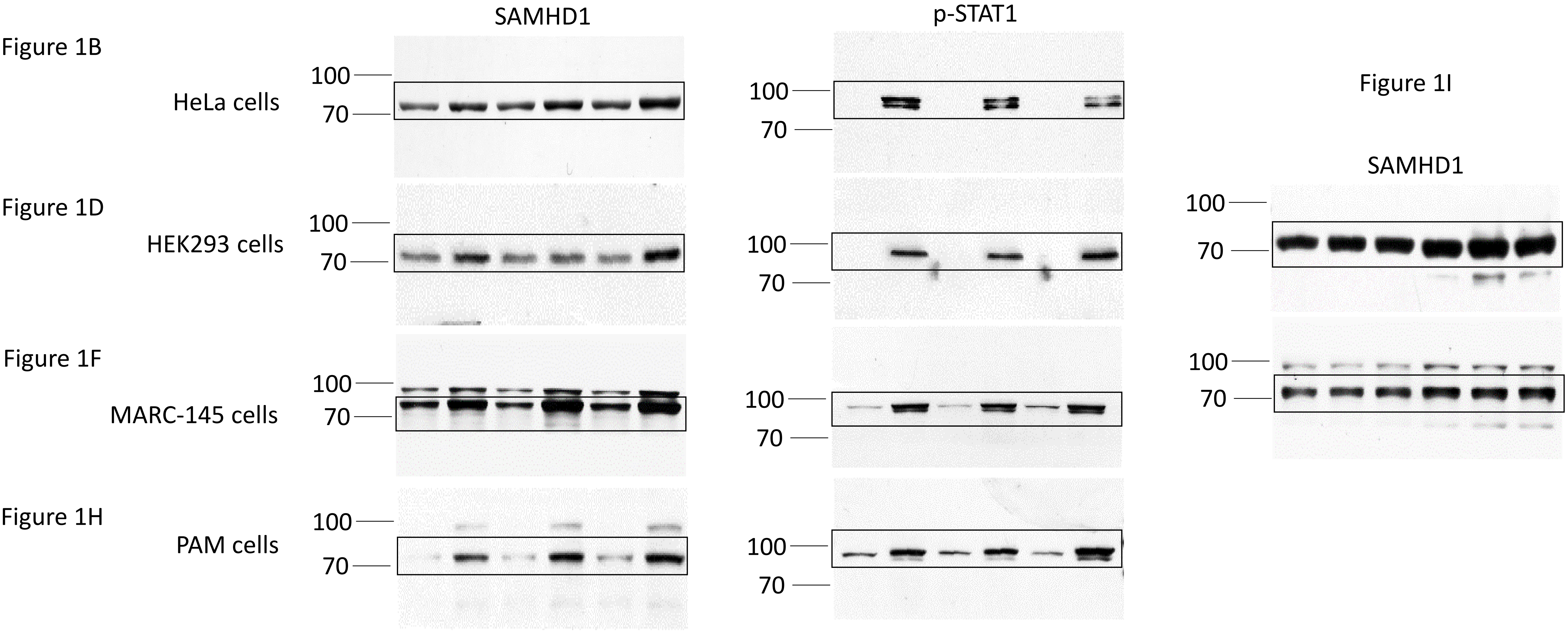
SAMHD1
p-STAT1
Figure 1B
HeLa cells
Figure 1D
HEK293 cells
Figure 1F
MARC-145 cells
Figure 1H
PAM cells
Figure 1I
SAMHD1
100
70
