# Supplementary material for: Interferon regulatory factor 3 is a key regulation factor for inducing the expression of SAMHD1 in antiviral innate immunity
Source: Sci Rep. 2016 Jul 14;6:29665. doi: 10.1038/srep29665 (PMC4944147; doi:10.1038/srep29665)

Interferon regulatory factor 3 is a key regulation factor for inducing the expression of SAMHD1 in antiviral innate immunity

Shen Yang, Yuan Zhan, Yanjun Zhou, Yifeng Jiang, Xuchen Zheng, Lingxue Yu, Wu Tong, Fei Gao, Liwei Li, Qinfeng Huang, Zhiyong Ma , Guangzhi Tong*

Shanghai Veterinary Research Institute, Chinese Academy of Agricultural Sciences, Shanghai, 200241, PR China

Figure 5B

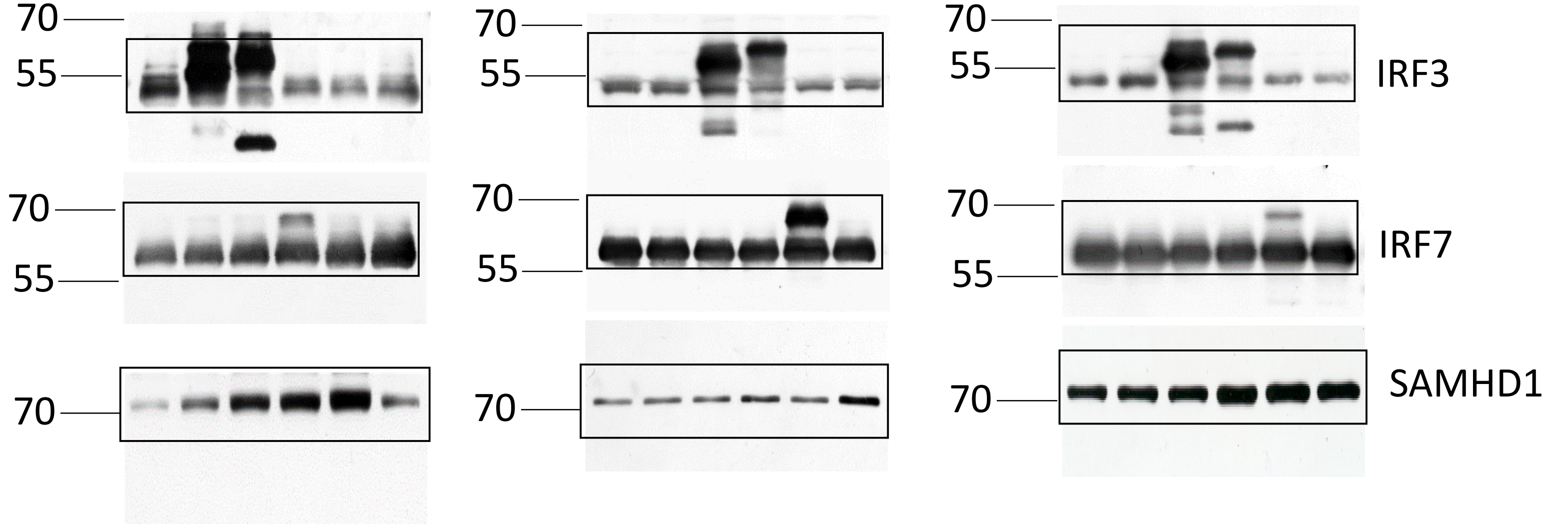

Figure 5D

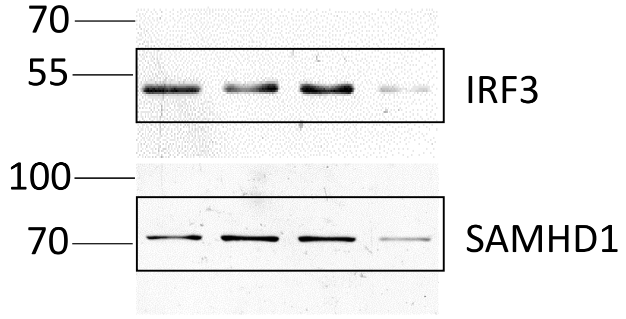

Figure 5E

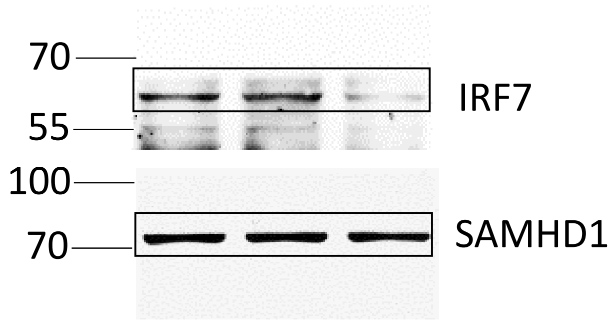

Figure 5F

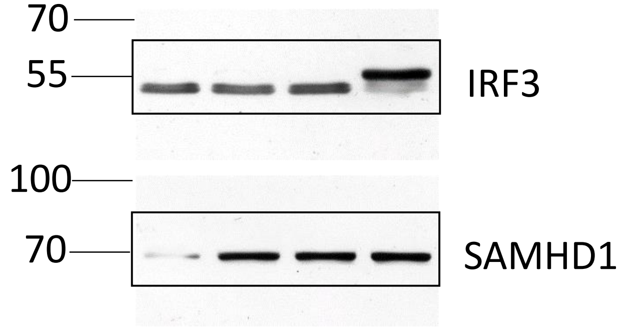

Supplement: Supplementary Figure 7 [file srep29665-s7.pdf]
