# Supplementary material for: Interferon regulatory factor 3 is a key regulation factor for inducing the expression of SAMHD1 in antiviral innate immunity
Source: Sci Rep. 2016 Jul 14;6:29665. doi: 10.1038/srep29665 (PMC4944147; doi:10.1038/srep29665)

Interferon regulatory factor 3 is a key regulation factor for inducing the expression of SAMHD1 in antiviral innate immunity

Shen Yang, Yuan Zhan, Yanjun Zhou, Yifeng Jiang, Xuchen Zheng, Lingxue Yu, Wu Tong, Fei Gao, Liwei Li, Qinfeng Huang, Zhiyong Ma , Guangzhi Tong*

Shanghai Veterinary Research Institute, Chinese Academy of Agricultural Sciences, Shanghai, 200241, PR China

Figure 6A

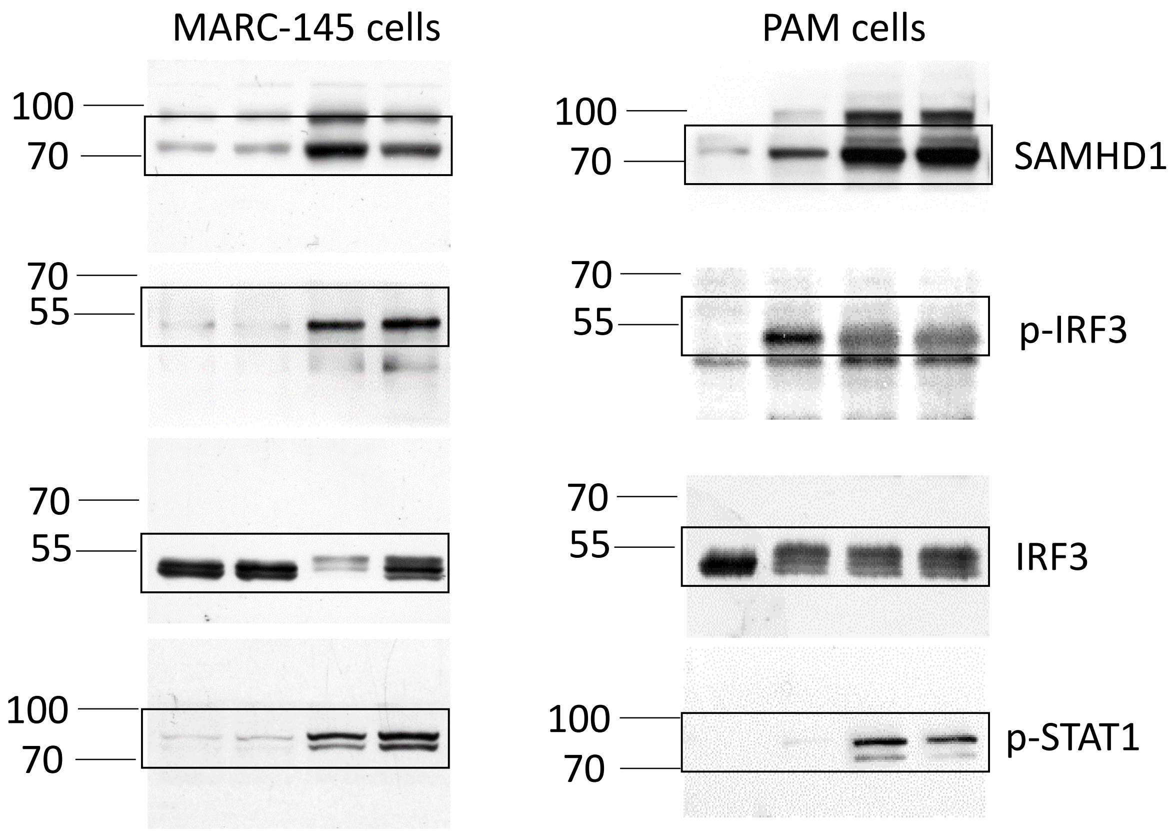

Figure 6B

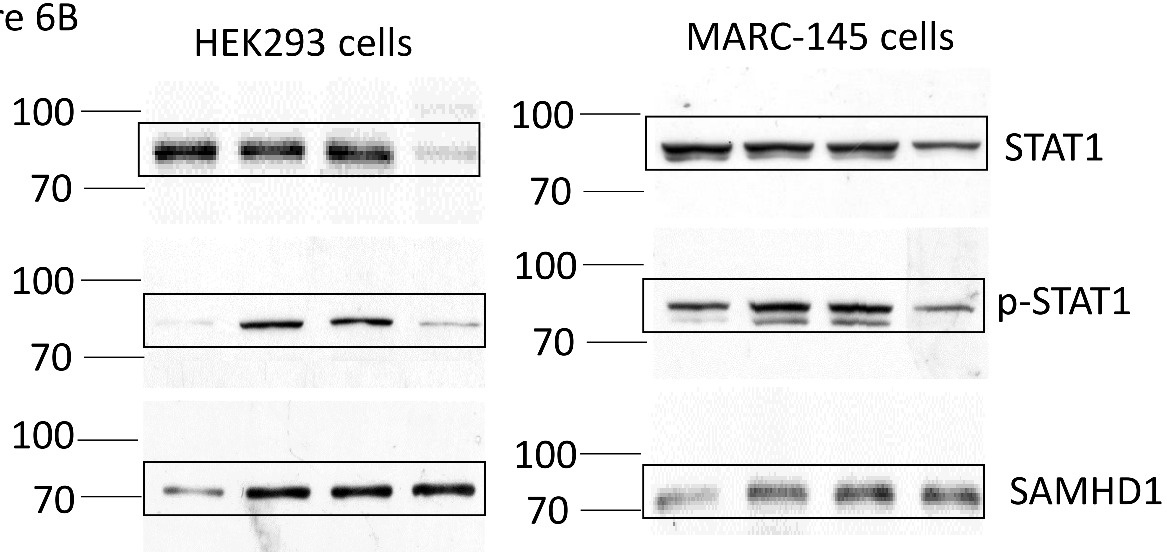

Figure 6C

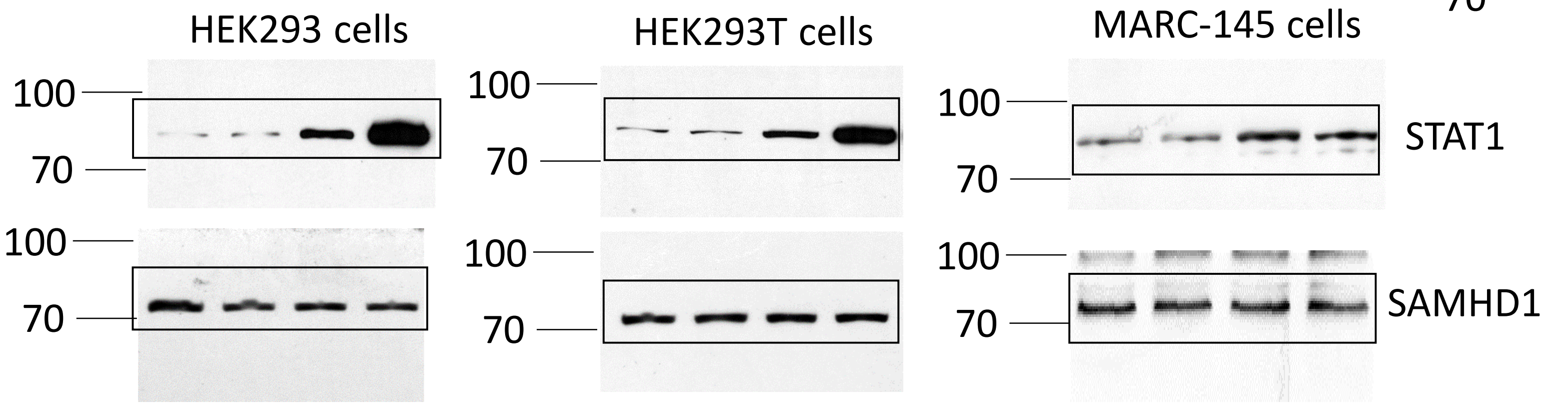

Figure 6D

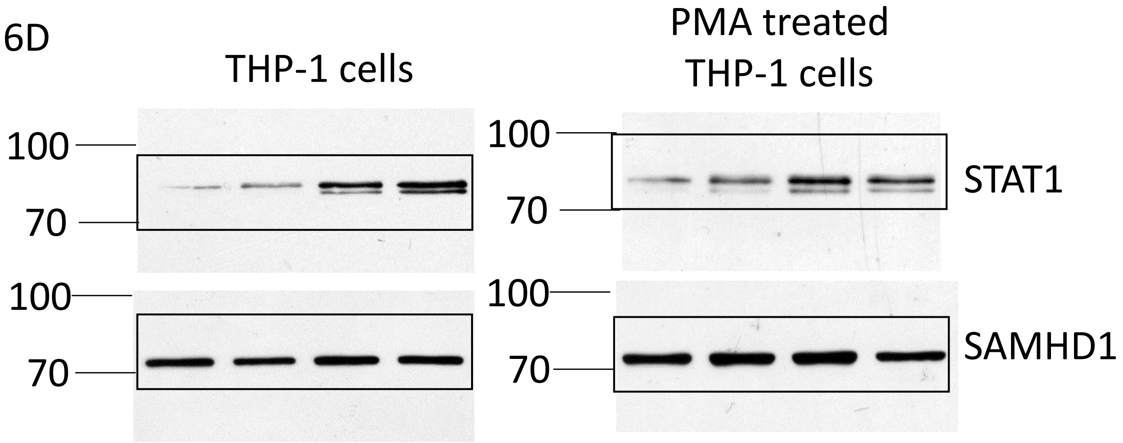

Supplement: Supplementary Figure 8 [file srep29665-s8.pdf]
